# Outbreaks of Disease Associated with Food Imported into the United States, 1996–2014^1^

**DOI:** 10.3201/eid2303.161462

**Published:** 2017-03

**Authors:** L. Hannah Gould, Jennifer Kline, Caitlin Monahan, Katherine Vierk

**Affiliations:** Centers for Disease Control and Prevention, Atlanta, Georgia, USA (L.H. Gould, J. Kline);; US Food and Drug Administration, College Park, Maryland, USA (C. Monahan, K. Vierk)

**Keywords:** foodborne illness, outbreaks, imported food, Salmonella, scombroid toxin, bacteria, food safety, United States, toxins

## Abstract

The proportion of US food that is imported is increasing; most seafood and half of fruits are imported. We identified a small but increasing number of foodborne disease outbreaks associated with imported foods, most commonly fish and produce. New outbreak investigation tools and federal regulatory authority are key to maintaining food safety.

Approximately 19% of food consumed in the United States is imported, including ≈97% of fish and shellfish, ≈50% of fresh fruits, and ≈20% of fresh vegetables (*1*). The proportion of food that is imported has increased steadily over the past 20 years because of changing consumer demand for a wider selection of food products and increasing demand for produce items year round (*1*).

The Centers for Disease Control and Prevention (CDC) defines a foodborne disease outbreak as the occurrence of >2 persons with a similar illness resulting from ingestion of a common food (*2*). Local, state, and territorial health departments report foodborne disease outbreaks to CDC through the Foodborne Disease Outbreak Surveillance System. The information collected for each outbreak includes etiology (confirmed or suspected on the basis of predefined criteria) (*2*), year, month, state, implicated food, and number of illnesses, hospitalizations, and deaths. Information is also collected on where implicated food originated. During 1973–1997, this information was reported anecdotally in the report’s comments section. During 1998–2008, “contaminated food imported into U.S.” was included as a location where food was prepared. Since 2009, the form has included a variable to indicate whether an implicated food was imported into the United States and the country of origin.

## The Study

We reviewed outbreak reports to identify outbreaks associated with an imported food from the inception of the surveillance system in 1973 through 2014, the most recent year for which data were available. We obtained additional data for some outbreaks (e.g., country of origin) from the US Food and Drug Administration (FDA) and the US Department of Agriculture Food Safety and Inspection Service.

We categorized implicated foods by using the schema developed by the Interagency Food Safety Analytics Collaboration (*3*). We grouped countries using the United Nations Statistics Division classification (*4*). We conducted a descriptive analysis of the number of outbreaks over time, by food category, and by region of origin.

During 1996–2014, a total of 195 outbreak investigations implicated an imported food, resulting in 10,685 illnesses, 1,017 hospitalizations, and 19 deaths. Outbreaks associated with imported foods represented an increasing proportion of all foodborne disease outbreaks where a food was implicated and reported (1% during 1996–2000 vs. 5% during 2009–2014). The number of outbreaks associated with an imported food increased from an average of 3 per year during 1996–2000 to an average of 18 per year during 2009–2014 (Figure).

**Figure:**
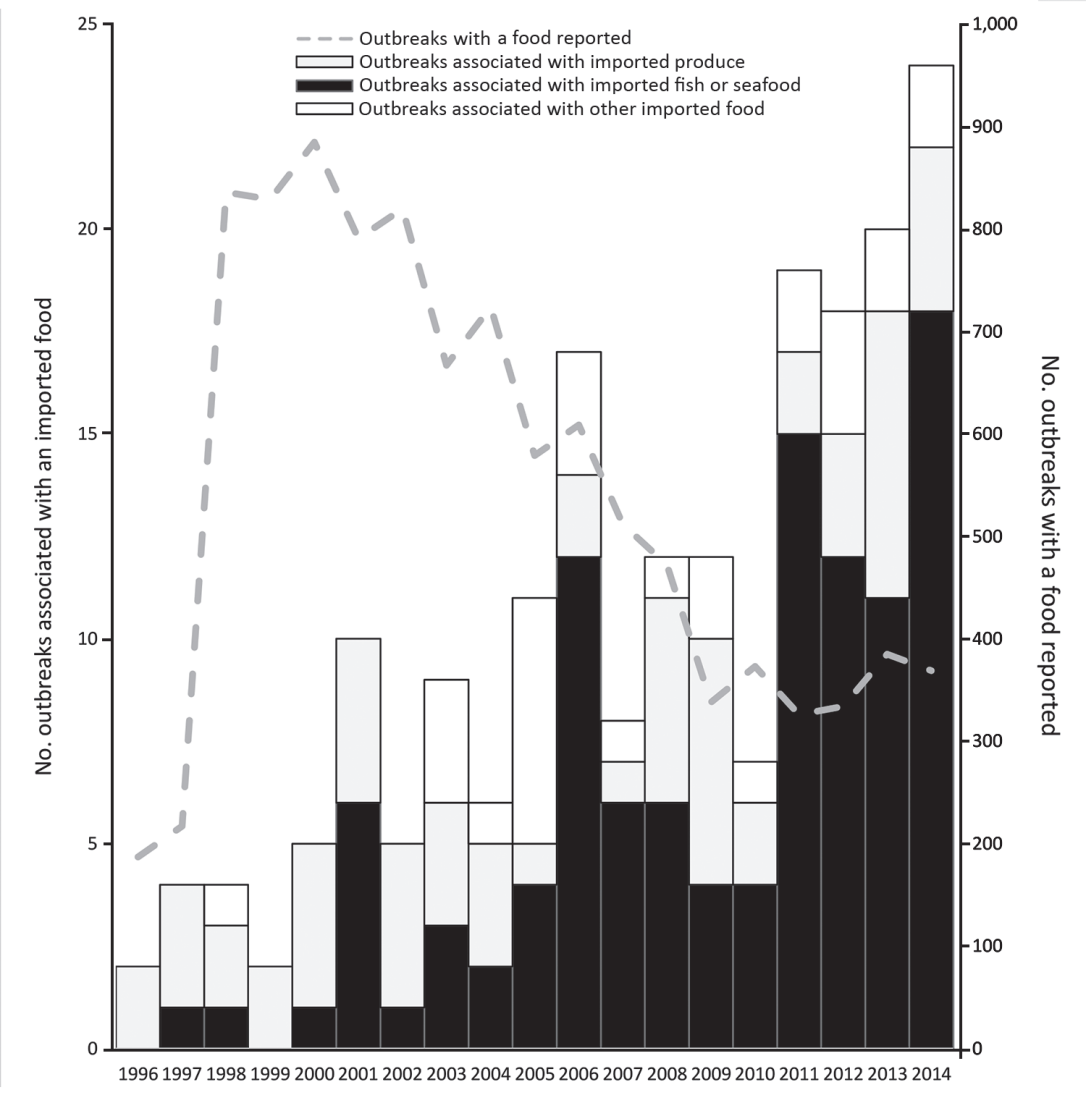
Number of outbreaks caused by imported foods and total number of outbreaks with a food reported, United States, 1996–2014. Reporting practices changed over time; 1973–1997, imported foods anecdotally noted in report comments; 1998–2008, “contaminated food imported into U.S.” included as a location where food was prepared; 2009–2014, reporting jurisdictions could indicate whether each food is imported (yes/no) and the country of origin.

The most common agents reported in outbreaks associated with imported foods were scombroid toxin and *Salmonella*; most illnesses were associated with *Salmonella* and *Cyclospora* (Table). Aquatic animals were responsible for 55% of outbreaks and 11% of outbreak-associated illnesses. Produce was responsible for 33% of outbreaks and 84% of outbreak-associated illnesses. Outbreaks attributed to produce had a median of 40 illnesses compared with a median of 3 in outbreaks attributed to aquatic animals. All but 1 of the outbreaks caused by scombroid toxin was associated with fish. Most of the *Salmonella* outbreaks (77%) were associated with produce, including fruits (n = 14), seeded vegetables (n = 10), sprouts (n = 6), nuts and seeds (n = 5), spices (n = 4), and herbs (n = 1).

**Table:** Outbreaks and illnesses caused by imported foods, by causative agent and food category, United States, 1996–2014*

Etiology	No. (%)	
	Outbreaks	Illnesses
Agent		
Scombroid toxin	57 (31)	192 (2)
* Salmonella*	52 (28)	4,421 (42)
Ciguatoxin	18 (10)	76 (0.7)
* Cyclospora*	11 (6)	3,533 (33)
Norovirus	10 (5)	131 (1)
*Escherichia coli* O157	6 (3)	116 (1)
* Shigella sonnei*	5 (3)	625 (6)
* Vibrio parahaemolyticus*	5 (3)	243 (2)
* Listeria monocytogenes*	4 (2)	67 (0.6)
Hepatitis A virus	4 (2)	1150 (11)
* Brucella*	3 (2)	11 (0.1)
Other†	9 (5)	38 (0.4)
Food category		
Aquatic animals		
Fish	88 (45)	830 (8)
Mollusks	17 (9)	350 (3)
Crustaceans	1 (0.5)	18 (0.2)
Other seafood	1 (0.5)	14 (0.1)
Land animals		
Dairy	12 (6)	140 (1)
Beef	1 (0.5)	29 (0.3)
Eggs	1 (0.5)	58 (0.5)
Game	1 (0.5)	2 (0)
Produce		
Fruits	22 (112)	3,450 (32)
Seeded vegetables	11 (6)	1,847 (17)
Sprouts	10 (5)	510 (5)
Vegetable row crops	7 (4)	1,241 (12)
Spices	4 (2)	530 (5)
Herbs	4 (2)	1,147 (11)
Other produce	2 (1)	154 (1)
Other plants		
Nuts and seeds	5 (3)	132 (1)
Oils and sugars	2 (1)	10 (0.1)
Grains and beans	1 (0.5)	89 (0.8)
Multiple etiology‡	5 (3)	134 (1)

*Causative agent data were available for 184 outbreaks involving 10,603 illnesses. Food category data were available for 195 outbreaks involving 10,685 illnesses. †Other agents implicated were tetrodotoxin (3 outbreaks) and *Campylobacter*, chaconine, *Paragonimus,* other virus, sulfite, and *Trichinella* (1 outbreak each). ‡Foods implicated were a chicken dish, crab cake, creampuff, beer, and a wheat snack (1 outbreak each).

Information was available on the region of origin for 177 (91%) outbreaks. Latin America and the Caribbean was the most common region implicated, followed by Asia (Technical Appendix). Thirty-one countries were implicated; Mexico was most frequently implicated (42 outbreaks). Other countries associated with >10 outbreaks were Indonesia (n = 17) and Canada (n = 11). Fish and shellfish originated from all regions except Europe but were most commonly imported from Asia (65% of outbreaks associated with fish or shellfish). Produce originated from all regions but was most commonly imported from Latin America and the Caribbean (64% of outbreaks associated with produce). All but 1 outbreak associated with dairy products involved products imported from Latin America and the Caribbean.

Outbreaks in this analysis were reported from 31 states, most commonly California (n = 30), Florida (n = 25), and New York (n = 16). Forty-three outbreaks (22%) were multistate outbreaks.

## Conclusions

The number of reported outbreaks associated with imported foods, although small, has increased as an absolute number and in proportion to the total number of outbreaks in which the implicated food was identified and reported. Although many types of imported foods were associated with outbreaks, fish and produce were most common. These findings are consistent with overall trends in food importation (*5*).

Many outbreaks, particularly outbreaks involving produce, were associated with foods imported from countries in Latin America and the Caribbean. Because of their proximity, these countries are major sources of perishable items such as fresh fruits and vegetables; Mexico is the source of about one quarter of the total value of fruit and nut imports and 45%–50% of vegetable imports, followed by Chile and Costa Rica. Similarly, our finding that many outbreaks were associated with fish from Asia is consistent with data on the sources of fish imports (*6*).

One quarter of the outbreaks were multistate, reflecting the wide distribution of many imported foods. Systems like PulseNet have helped to improve detection and investigation of multistate outbreaks, resulting in an increased number of multistate outbreaks (*7*,*8*). The increasing number of outbreaks involving globally distributed foods underscores the need to strengthen regional and global networks for outbreak detection and information sharing. The importance of having standard protocols for molecular characterization of isolates and systems for rapid traceability of implicated foods to their source was illustrated during the investigation of a listeriosis outbreak linked to Italian cheese imported into the United States in 2012 (*9*). Newer tools like whole genome sequencing can also help to generate hypothetical transmission networks and in some instances facilitate traceback of foods to their origin (*10*). Moreover, new tools that aid visualization of supplier networks facilitate the investigation of outbreaks involving the increasingly complex global economy (*11*).

Nearly all of the outbreaks involved foods under FDA jurisdiction. Only a small proportion of FDA-regulated foods are inspected upon entry into the United States. New rules under the Food Safety Modernization Act of 2011, including the Preventive Controls Rule for Human Food, Produce Safety Rule, Foreign Supplier Verification Program, and Accreditation of Third Party Auditors, will help to strengthen the safety of imported foods by granting FDA enhanced authorities to require that imported foods meet the same safety standards as foods produced domestically (*12*).

Although data collection has improved in recent years, these findings might underestimate the number of outbreaks associated with imported foods because the origin of only a small proportion of foods causing outbreaks is reported. Similarly, because of how data are collected and reported, the relative safety of imported and domestically produced foods cannot be compared. Because of changes in surveillance and changing import patterns, changes over time should be interpreted cautiously.

Our findings reflect current patterns in food imports and provide information to help guide future outbreak investigations. Prevention focused on the most common imported foods causing outbreaks, produce and seafood, could help prevent outbreaks. Efforts to improve the safety of the food supply can include strengthening reporting by gathering better data on the origin of implicated food items, including whether imported and from what country.

Technical Appendix Region and country of origin of imported foods implicated in outbreaks, by food category, United States, 1996–2014.
